# Function of sesquiterpenes from *Schizophyllum commune* in interspecific interactions

**DOI:** 10.1371/journal.pone.0245623

**Published:** 2021-01-15

**Authors:** Sophia Wirth, Katrin Krause, Maritta Kunert, Selina Broska, Christian Paetz, Wilhelm Boland, Erika Kothe

**Affiliations:** 1 Microbial Communication, Institute of Microbiology, Friedrich Schiller University Jena, Jena, Germany; 2 Bioorganic Chemistry, Max Planck Institute for Chemical Ecology, Jena, Germany; 3 Research Group Biosynthesis/NMR, Max Planck Institute for Chemical Ecology, Jena, Germany; USDA Forest Service, UNITED STATES

## Abstract

Wood is a habitat for a variety of organisms, including saprophytic fungi and bacteria, playing an important role in wood decomposition. Wood inhabiting fungi release a diversity of volatiles used as signaling compounds to attract or repel other organisms. Here, we show that volatiles of *Schizophyllum commune* are active against wood-decay fungi and bacteria found in its mycosphere. We identified sesquiterpenes as the biologically active compounds, that inhibit fungal growth and modify bacterial motility. The low number of cultivable wood inhabiting bacteria prompted us to analyze the microbial community in the mycosphere of *S*. *commune* using a culture-independent approach. Most bacteria belong to *Actinobacteria* and *Proteobacteria*, including *Pseudomonadaceae*, *Sphingomonadaceae*, *Erwiniaceae*, *Yersiniaceae* and *Mariprofundacea* as the dominating families. In the fungal community, the phyla of ascomycetes and basidiomycetes were well represented. We propose that fungal volatiles might have an important function in the wood mycosphere and could meditate interactions between microorganisms across domains and within the fungal kingdom.

## Introduction

Wood contains a high amount of lignin and is therefore difficult to decompose. Therefore, colonization with saprotrophic fungi is considered as the key driver for wood decomposition. The respective basidiomycetes are able to degrade lignin by secretion of lignocellulolytic enzymes, e.g. laccases and other peroxidases [1, 2]. Furthermore, bacteria have been reported to colonize wood [3] and to contribute either indirectly to wood decay by competition with fungi or directly by decomposing cellulose and hemicelluloses [4], and it has been indicated that bacteria are also involved in lignin degradation [5]. Saprophytic fungi and bacteria are in competition for the same resources and it is likely that antagonistic interactions influence the community structure, which in turn has an effect on the rate of wood decomposition and nutrient cycling [6].

Wood decomposition involves chemical [7], as well as structural changes [8] affecting the permeability through formation of pores, and it provides a heterogenous environment for the colonization of different niches. Metabolites such as volatiles and diffusible organic compounds can diffuse through these water or gas filled pores, enabling the exchange of substances over large distances [9]. In addition to mutualistic or commensal interactions, the result of competition among wood-inhabiting fungi or with bacteria can be scored as, e.g., deadlock or replacement [2, 10]. These antagonistic reactions are accompanied by the production of extracellular enzymes and secondary metabolites (like the dark-colored pigment indole) suggesting a specific defense program against antagonists [11, 12].

Volatile organic compounds (VOCs) are generally considered infochemicals mediating inter- and intraspecies interactions [13]. Fungi emit a complex mixture of VOCs including alcohols, benzenoids, hydrocarbons, aldehydes, alkanes, alkenes, acids, esters, terpenes and ketones [14] contributing to their characteristic odor. Some VOCs are emitted from different fungal species, but some compounds are unique for specific strains and might be applicable as biomarkers for the identification of fungi [15]. The biological as well as ecological function of VOCs as indicators of fungal growth, their role as semiochemicals for insects, in "mycofumigation" and as mediators of interactions between organisms within and across different ecological niches has been discussed [13]. However, volatile profiles are considered as dynamic and their composition depend on the growth substrate, pH, temperature and culture age [16, 17].

The emission of fungal volatiles linked to interspecific interactions and defense processes could be shown during interactions, e.g. between *Hypholoma fasciculare* and *Resinicum bicolor*, where both fungi produce new VOCs during co-cultivation [16]. Mostly, sesquiterpenes were identified, some of which show antifungal activity or affect mycelial morphology [9, 10, 18]. In addition, pigment production is often connected to sesquiterpene release which strengthens their potential role in defense responses [16].

The saprophytic fungus *Schizophyllum commune* releases ketones, sesquiterpenes and ethanol [19, 20]. Interestingly, the production of sesquiterpenes can be linked to its sexual life cycle [21]. The VOC profile of *S*. *commune* is biologically active against plant pathogenic fungi presumably involving ethanol and the sesquiterpene β-bisabolol [20]. Therefore, sesquiterpene-overproducing mutants of *S*. *commune* (*thn* mutants) were examined [22]. The high amount of (-)-(1*R*,2*S*)-β-bisabolol released by these strains allows studying the function of sesquiterpenes as antimicrobial metabolites. Here, we characterized the natural fungal and bacterial community by a DNA-dependent approach to gain a deeper understanding of signaling in the mycosphere of *S*. *commune*. We tested the antimicrobial potential of *S*. *commune* volatiles in different life stages involving unmated monokaryon, fertile dikaryon, and included sesquiterpene over-producing strains. To link effects of VOCs to interactions occurring in nature, the mycelial growth of tester strains likely to co-occurring in timber was tested. In addition, we investigated the potential of *S*. *commune* to inhibit bacterial growth, modify bacterial motility and to induce morphological changes *via* volatile emission.

## Materials and methods

### Strains and culture conditions

All fungal and bacterial strains used in this study were provided by the Jena Microbial Resource Collection (JMRS, Jena, Germany). The *S*. *commune* strains 4–39 (FSU 2896) and 12-43x4-39 (FSU 11842) were used, and plant colonizing fungi and bacteria were chosen (S1 Table in S1 File). One strain was isolated from the phyllosphere, two derived from the rhizosphere and four are saprophytes causing white rot on wood. The *S*. *commune thn* strains (4–39 *thn*, H4-8 *thn* and W22 *thn*) resulting from transposition into the *thn1* gene (ID 83983) were used to allow for scoring sesquiterpene over-production effects. The *thn1* gene encodes a regulator for G-protein signaling [23].

Fungi were initially cultivated on complex yeast extract medium (CYM) with 1.8% agar at 28°C for several days [24]. Sesquiterpene extraction was performed from *S*. *commune* liquid cultures grown for 3 days at 150 rpm and 28°C. Bacterial strains were cultivated and maintained on Standard I (StdI; Carl Roth, Karlsruhe, Germany) and incubated for 1–2 days at 28°C. Bacterial swarming activity was determined on soft StdI containing 0.6% agar [25].

### Quantification and extraction of fungal sesquiterpenes

Fungal volatile analysis of *S*. *commune* surface cultures was carried out using solid phase microextraction (SPME). Headspace volatiles were collected over a period of 1 hour from *S*. *commune* cultivated on CYM in polystyrene Petri dishes. The SPME fibers and the holder were obtained from Supelco (Bellefonte, PA, USA). A divinylbenzene-carboxen-polydimethylsiloxane (50/30 μm DVB/CAR/PDMS) fiber was used. The fibers were first conditioned according to the manufacturer´s instructions. Prior to sampling, the Petri dish was opened and accumulated volatiles were removed by a gently stream of nitrogen. The SPME fiber was inserted into the volatile collection unit through a hole in the Petri dish before enclosing it with polyethylene terephthalate foil (PET; Toppits Bratschlauch, Minden, Germany).

For quantification of sesquiterpenes, a closed loop stripping system was used as previously described [26]. In brief, the closed loop stripping system consists of two stainless steel tubes placed over a fungal culture. The collection unit was enclosed with polyethylene terephthalate foil and the tubes were connected to a rotary vane pump (model G 12/02 EB, Denver, Fürstenfeldbruck, Germany) equipped with a stainless steel housing for the trap [27]. The volatiles emitted by the fungi were continuously collected over a period of 3 hours on small charcoal traps (1.5 mg of charcoal, CLSA-Filter; Gränicher, Daumazan sur Arize, France) and eluted in two steps with 50 μL (2 x 25 μl; sample 1) and 150 μl (sample 2) dichloromethane containing an internal standard (dodecane, 50 μg ml^-1^; Merck, Darmstadt, Germany). Measurement was performed using five biological replicates for each strain.

For gas chromatography-mass spectrometry (GC-MS) analysis, a TRACE MS device (Thermo-Finnigan, Bremen, Germany) equipped with a ZB5 column (15 m, 0.25 mm I.D, 0.25 μm film thickness and 10 m guard column; Phenomenex, Aschaffenburg, Germany) was used. Mass spectra were recorded in electron impact (EI) mode at 70 eV, covering a mass range from m/z 33–450 Da. Volatiles were eluted under programmed conditions: 45°C (2 min isotherm), followed by heating at 10°C min_-1_ to 200°C and at 30°C min_-1_ to 280°C, using helium (1.5 ml min_-1_) as the carrier gas. The GC injector (split ratio 1:10), transfer line and ion source were set at 220, 280 and 200°C, respectively. Bisabolene isomers were identified by comparing their Kováts retention indices [28] with those from reference databases and a synthetic bisabolene mix standard by injecting an *n*-alkane standard solution C_8_-C_20_ in hexane (Fluka, Steinheim, Germany; see S1 Fig in S1 File).

**Fig 1:**
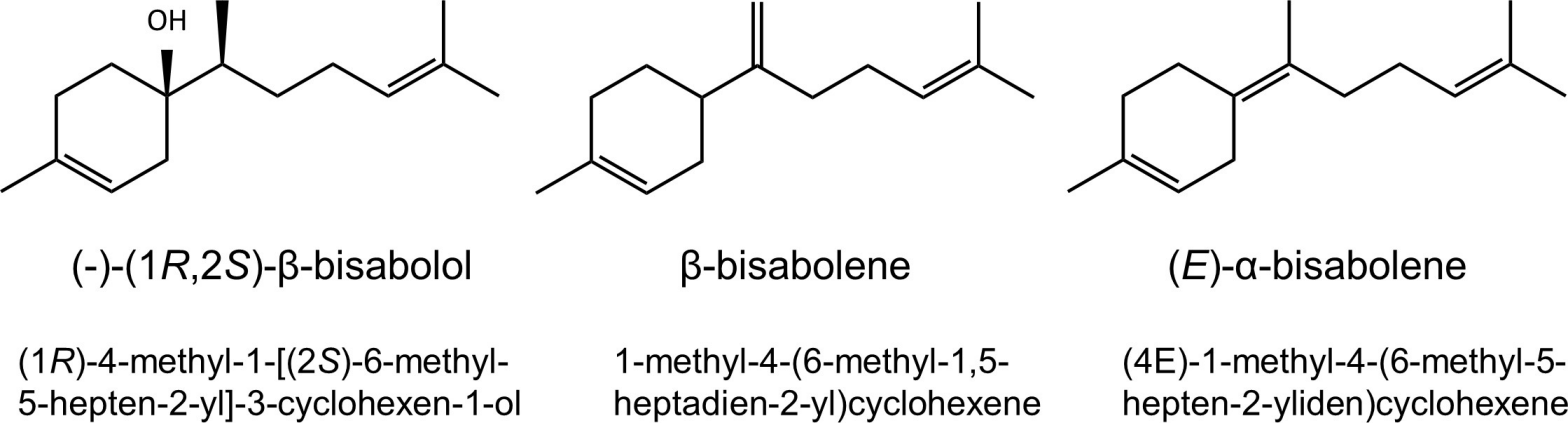
Structures and names of the major sesquiterpenes of *S*. *commune*.

The sesquiterpenes (1*R*)-4-methyl-1-[(2*S*)-6-methyl-5-hepten-2-yl]-3-cyclohexen1-ol (= (-)-(1*R*,2*S*)-β-bisabolol, 1-methyl-4-(6-methyl-1,5-heptadien-2-yl)cyclohexene (= β-bisabolene) and (4*E*)-1-methyl-4-(6-methyl-5-hepten-2-yliden)cyclohexene (= (*E*)-γ-bisabolene) were quantified using α-bisabolol (Alfa Aesar, Karlsruhe, Germany) as calibration standard and dodecane as internal standard (concentration 50 μg ml^-1^ compare Fig 1). A calibration range from 5 ng to 1000 ng (5, 10, 20, 40, 50, 60, 80, 100, 150, 200, 250, 500, 750, 100 μg ml^-1^ concentration–injected 1 μl into the GC-MS–linear calibration R^2^ = 0.997) of α-bisabolol was used for (-)-(1*R*,2*S*)-β-bisabolol. The amount of β-bisabolene and (*E*)-γ-bisabolene was estimated from different *S*. *commune thn* strains using five replicates according to the calibration of α-bisabolol (calibration range 5 ng to 50 ng).

For bioassays, (-)-(1*R*,2*S*)-β-bisabolol extracted by hydrodistillation from *S*. *commune* H4-8 *thn* and *S*. *commune* W22 *thn* liquid cultures and a commercial available mix of different bisabolene isomers was used (CAS: 495-62-5, Alfa Aesar, Karlsruhe, Germany). GC-MS analysis ensured that the commercially available bisabolene mix is comparable with bisabolene emission by *S*. *commune thn* mutants and therefore suitable for volatile bioassays (S1 Fig in S1 File). Hydrodistillation was performed with 25–40 g mycelium, which has been ground to a fine powder in liquid nitrogen and subsequently dissolved in 500 ml *A*. *dest*. (Clevenger-type apparatus, Hofmann, Staudt, Germany). The steam passes to the condenser where the volatile compounds were collected in 1 ml *n*-hexane (Rotisolv HPLC, Carl Roth, Karlsruhe, Germany). After 3 h the extraction was stopped. The *n*-hexane was removed by a gentle stream of nitrogen and the (-)-(1*R*,2*S*)-β-bisabolol was purified by silica gel chromatography with pentane-diethyl ether (9:1; v:v). (-)-(1*R*,2*S*)-β-bisabolol showed a Rf value of 0.28. The procedure was performed several times to obtain sufficient material for bioassays (0.01% average yield of volatile substances; 37% average yield of (-)-(1*R*,2*S*)-β-bisabolol after purification. Product identity was verified by nuclear magnetic resonance (NMR) spectroscopy and GC-MS yielding a purity of more than 95% for (-)-(1*R*,2*S*)-β-bisabolol as described in [22]. NMR spectra (^1^H, ^13^C DEPTQ, ^1^H-^1^H COSY, ^1^H-^13^C HSQC, ^1^H-^13^C HMBC and ^1^H-^1^H ROESY) were measured on a Bruker Avance III HD 700 spectrometer, equipped with a cryoplatform and a 1.7 mm TCI CryoProbe (Bruker BioSpin GmbH, Rheinstetten, Germany). A NMR tube of 1.7 mm outer diameter was used and chemical shifts were referenced to TMS at δH/δC 0 ppm. Data acquisition and processing were accomplished using the software TopSpin 3.2 (Buker BioSpin).

The statistical analyses were performed with unpaired Student’s t-test.

### Exposure to fungal volatiles

To study the effect of *S*. *commune* volatiles, fungi and bacteria were cultivated in two-compartment Petri dishes (Greiner, Frickenhausen, Germany). The physical barrier in these Petri dishes excludes physical interaction, but allows for signaling exclusively *via* the gas phase. Fungal agar plugs of 2 mm diameter were used for inoculation on CYM plates closed with Parafilm (Bemis, Neenah, USA) prior to incubation for 8 days at 28°C. Control plates were prepared similarly using sterile growth medium in the second compartment. Analysis of fungal growth was documented using the SPOT advanced software (version 4.6, Diagnostic instruments, Houston, USA). To study the effect of *S*. *commune* volatiles on bacteria, *S*. *commune* was pre-cultivated on CYM for 5 days at 28°C before bacterial strains were inoculated after growth for 1–2 days in StdI at 120 rpm and 28°C, washing in 0.8% NaCl, and inoculated at an optical density OD_600_ 0.6 diluted 10^−5^ in 0.8% NaCl. Incubation took place for 1–2 days at 28°C with five biological replicates. The activity of *S*. *commune* volatiles was determined by comparing the cfu ml^-1^ of bacteria exposed to fungal volatiles to that of bacteria exposed to sterile CYM. Bacterial swarming activity was determined on StdI soft agar [25].

The effects of sesquiterpenes on fungal and bacterial growth were evaluated using two-compartment Petri dishes with indicator fungi or bacteria in one compartment on CYM or StdI, respectively. The second compartment was equipped with sterile paper disks (6 mm diameter; Carl Roth, Karlsruhe, Germany) and loaded with the selected sesquiterpene bisabolene (30 μg; Alfa Aesar, Karlsruhe, Germany) or (-)-(1*R*,2*S*)-β-bisabolol (2 mg) corresponding to a final concentration of 0.6 mg l^-1^ bisabolene and 40 mg l^-1^ (-)-(1*R*,2*S*)-β-bisabolol. Pure sesquiterpenes were dissolved in *n*-hexane (GC-ultra grade, Carl Roth, Karlsruhe, Germany) to obtain stock solutions. Calculation assumed the complete compound evaporation from the filter paper and corresponds to a 6-day-cocultivation period. As a control, *n*-hexane (GC-ultra grade, Carl Roth,Karlsruhe, Germany) was used. The sealed plates were incubated for several days at 28°C. Bacterial swarming motility assayed on StdI soft agar. The activity of the pure compounds was determined by comparing the diameter of bacterial swarming to that of bacteria exposed to *n*-hexane. Data are shown as average values with standard deviation. The statistical analyses were performed with unpaired Student’s t-test.

### Community analyses

Wood was sampled from *Salix* sp. in Jena, Germany (N 50° 55' 16.4”, E 11° 34' 54.1”, altitude 149 m) in 2016 adjacent to fruiting bodies of *S*. *commune*. The upper 3 cm of wood were removed; the surface sterilized with ethanol, and a sterile drill was used. The core was stored at 4°C for further analysis. DNA extraction was performed from 1 g wood (fresh weight), which has been flash-frozen in liquid nitrogen and ground to a fine powder using the MoBio Soil DNA Extraction Kit (MoBio, Carlsbad, USA). Extraction was performed three times and DNA was pooled to ensure a representative wood community. DNA was purified using the OneStep PCR Inhibitor Removal Kit (Zymo Research, Irvine, USA) and sequenced (MR DNA, Shallowater, USA). The 16S rDNA hypervariable regions V1 to V3 were addressed using the primer pair whoi341 (5‘-CCT ACG GGN GGC WGC AG) and whoi785 (5’-GAC TAC HVG GGT ATC TAA TCC) for bacteria and ITS1 using the primers ITS1F (5’-CTT GGT CAT TTA GAG GAA GTA A) and ITS2 (5'-GCT GCG TTC TTC ATC GAT GC) for fungi [29]. Data analysis was performed using the MR DNA analysis pipeline, including joining of sequences after removing short sequences (<150 bp). Operational taxonomic units (OTUs) were defined by 97% similarity. The Illumina MiSeq data and relevant sequences can be found under BioProject PRJNA662029 (NCBI at http://www.ncbi.nlm.nih.gov). Rarefaction curves, Shannon diversity index and Simpson diversity index were generated using Past 3.15 [30].

### Strain isolation and identification

A microbial suspension was prepared by dissolving 1 g finely powdered wood in 9 ml 0.8% NaCl and plating on StdI or TSB medium [3] containing 100 μg ml^-1^ cycloheximide (Carl Roth, Karlsruhe, Germany) for bacteria and CYM for isolation of fungi. All plates were incubated at 28°C for 2–7 days. Morphologically different strains were selected and identified after DNA extraction (DNeasy Blood and Tissue Kit, Qiagen, Hilden, Germany) for bacteria and Cenis (1992) for fungi [31], 16S rDNA (primers fD1: 5‘-AGA GTT TGA TCC TGG CTC AG and rP2: 5‘-ACG GCT ACC TTG TTA CGA CTT) or ITS (primers ITS1 and ITS4) amplification with Dream Taq Polymerase (Thermo Fisher, Waltham, USA) using manufacturer recommendations and sequencing (GATC, Konstanz, Germany). Identification was achieved using BlastN (NCBI at http://www.ncbi.nlm.nih.gov/). Sequences have been deposited in GenBank (http://www.ncbi.nlm.nih.gov/genbank) under accession numbers MT975277, MT975278, MT975284, MT975430 and MT984298.

## Results

### Bacterial and fungal community in wood degradation

Wood samples were collected from *Salix* sp. showing early signs of wood degradation adjacent to a fruiting body of *S*. *commune*. Only a small proportion was cultivated on StdI (1.71 x 10^11^ cfu per g of wood) and TSB medium (3.54 x 10^6^ cfu per g of wood). Successful isolation was achieved for Gammaproteobacteria, with the phyla *Pseudomonadaceae* and *Rhodanobacteraceae*, or Actinobacteria (mainly *Microbacteriaceae* and *Intrasporangiaceae;* S2 Table in S1 File). Aside from *S*. *commune*, no other fungal species could be cultivated confirming both its presence and its suppression of other wood rotting fungi (S3 Table in S1 File). The altogether low numbers of cultivatable species suggested the occurrence of interspecies competition in wood, which restricts bacterial or (other) fungal growth and might lead to the isolation of presumably adapted organisms.

To gain a better insight into the community structure, in addition to the necessary cultivation approach yielding strains for subsequent testing of volatile effects on co-occurring organisms, the fungal and bacterial community composition was analyzed in a culture-independent approach. A total of 160,145 high quality filtered bacterial and 35,956 high quality filtered fungal read pairs were obtained from wood samples. However, the rarefaction curves did not approach saturation, which indicates that the diversity is high, and yet undetected microorganisms are present in the community (S2 Fig and S4 Table in S1 File). Sequence analysis at 97% cut-off revealed 162 different bacterial OTUs. Of these, OTUs represented by more than 17 reads (0.01%) of the total community showed dominance of Proteobacteria (69.3%), with especially Alpha*-* and Gammaproteobacteria present (24.5% and 27.6%, respectively; *Pseudomonadaceae*, *Sphingomonadaceae*, *Erwiniaceae*, *Yersiniaceae* and *Mariprofundacea* were well represented). A high number of reads belonged to Actinobacteria (10.4%; Table 1, S2 Table in S1 File).

**Table 1:** Bacterial community composition in the mycosphere of *S*. *commune* presented as relative abundance (reads) and diversity (number of OTUs) at family level.

Family	Number of OTUs	Reads	Reads (%)
***Pseudomonadaceae***	10	77157	48.34
***Yersiniaceae***	2	46054	28.85
***Erwiniaceae***	3	22454	14.07
***Enterobacteriaceae***	4	5332	3.34
***Mariprofundaceae***	2	1016	0.64
***Sphingomonadaceae***	9	623	0.39
***Oxalobacteraceae***	5	430	0.27
***Sinobacteraceae***	4	400	0.25
***Sphingobacteriaceae***	3	328	0.21
***Hyphomicrobiaceae***	7	315	0.20
***Xanthomonadaceae***	4	304	0.19
***Micrococcaceae***	1	285	0.18
***Chitinophagaceae***	4	242	0.15
***Micromonosporaceae***	1	242	0.15
***Burkholderiaceae***	2	211	0.13

Of the total OTUs, 47.2% (76,736 reads) belonged to known lignocellulolytic genera (*Bacillus*, *Burkholderia*, *Cupriavidus*, *Paenibacillus*, *Mycobacterium*, *Methylobacterium*, *Novosphingobium*, *Pseudomonas* and *Variovorax*). Among the fungi, ascomycetes and basidiomycetes dominated (48.15 and 27.78%, respectively). Most of these OTUs were classified as basidiomycetous Agaricomycetes, or ascomycetous Sordariomycetes and Dothideomycetes. *S*. *commune* was confirmed to dominate with 95.2%. This was followed by the ascomycete *Coniochaeta* (*Lecythophora)* sp. (S3 Table in S1 File). With respect to different life styles, saprophytic fungi comprised 32% of the sequences, symbiotic fungi 30% and phytopathogenic fungi 16% (S3 Fig in S1 File).

### Effect of *S*. *commune* volatiles on fungal and bacterial growth

The low number of cultivatable, presumably adapted organisms, may bias the investigation of interspecies interaction. Moreover, secondary metabolite profiles of *S*. *commune* change during life stage, with specifically dikaryons producing a variety of VOCs of different chemical classes [21]. To investigate the effect of *S*. *commune* volatiles on the growth of other basidiomycetes (*Pleurotus ostreatus*, *Ganoderma lucidum*, *Flammulina velutipes* and *Kuehneromyces mutabilis)* and bacteria (*Pseudomonas fluorescens*, *Erwinia amylovora* and *Serratia marcescens*), co-cultivation in two-compartment Petri dishes excluding physical contact was performed. In these experiments, *S*. *commune* wildtype monokaryon and dikaryon did not influence the growth of *G*. *lucidum*, *F*. *velutipes* and *K*. *mutabilis*, and only slightly inhibited *P*. *ostreatus* (S4 Fig in S1 File).

**Fig 2:**
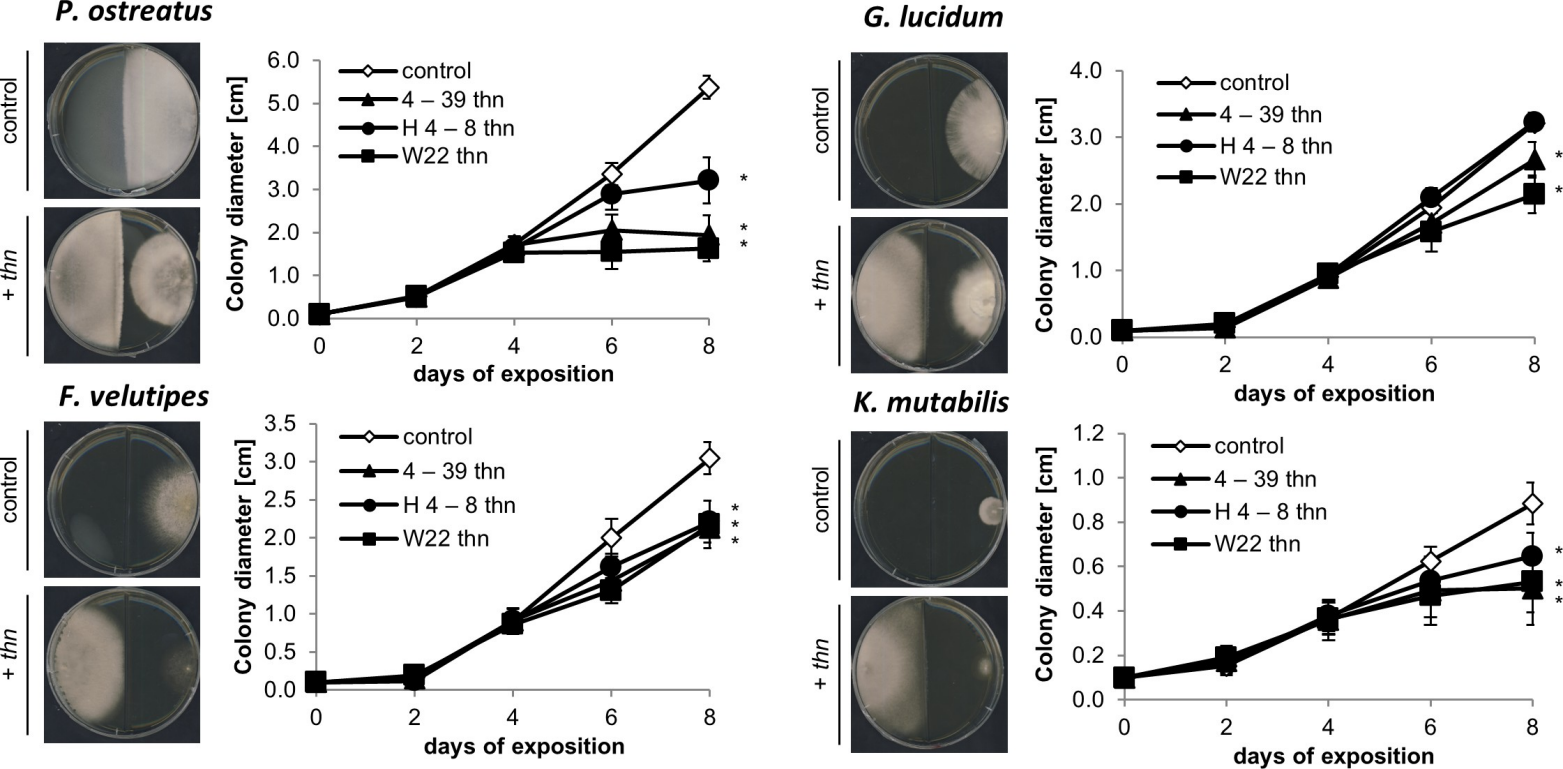
Effect of *S*. *commune* Volatile Organic Compounds (VOCs) on the growth of indicator fungi. *P* < 0.0001, n = 10.

Since *S*. *commune thn* mutants show a similar VOCs pattern like a S. *commune* dikaryon with increased production of sesquiterpenes including (-)-(1*R*,2*S*)-β-bisabolol, β-bisabolene and (*E*)-γ-bisabolene, a more intense response might be expected. Indeed, all tested fungi responded to volatiles of three different *thn* mutants with a strong mycelial growth reduction (Fig 2). This effect was least pronounced with *G*. *lucidum* which significantly responded only to two *thn* strains, *S*. *commune* 4–39 *thn* and *S*. *commune* W22 *thn*.

**Fig 3:**
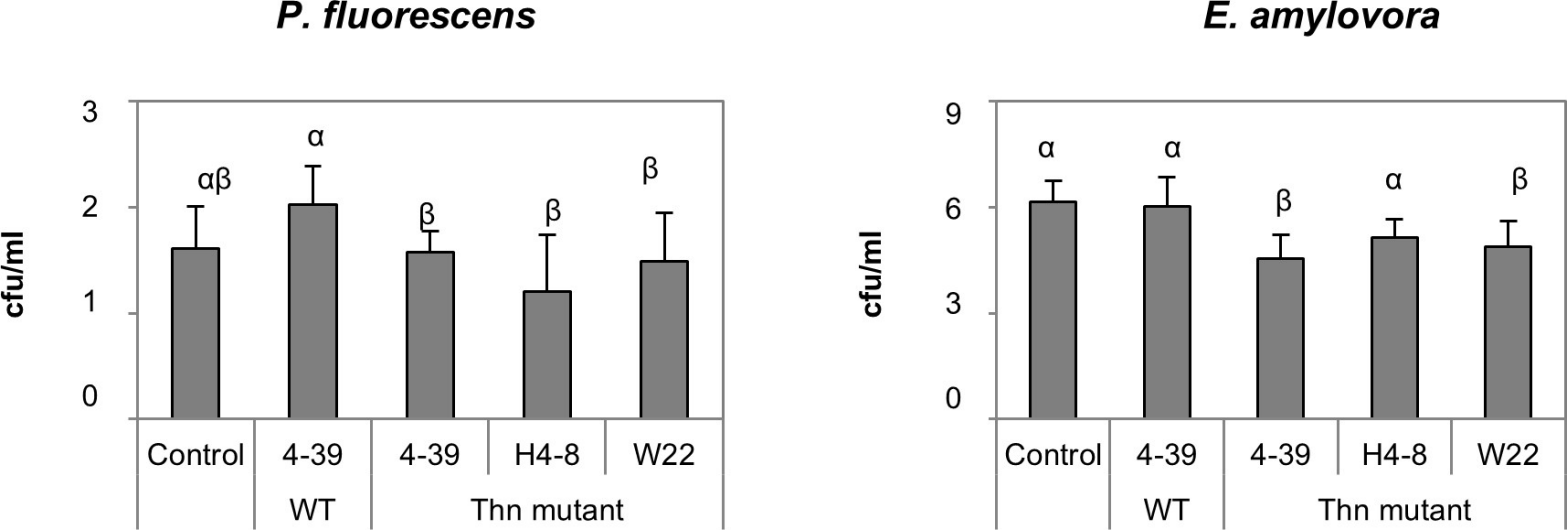
Effect of volatiles produced by *S*. *commune* wildtype and *thn* mutant strains on the growth of indicator bacteria. Data are presented as viable cell numbers (cfu ml^-1^) in 10^8^; *P* < 0.05, n = 6.

**Fig 4:**
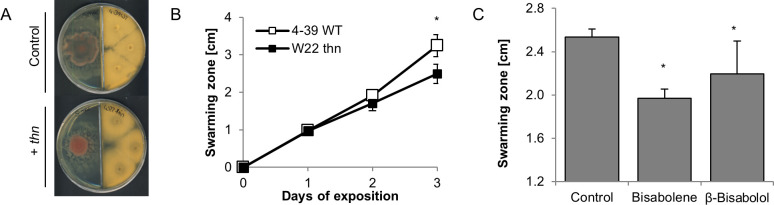
Effect of sesquiterpenes on *S*. *marcescens* swarming motility. (A) Setup of experiment, (B) effect of fungal co-cultivation and (C) exposure to (-)-(1*R*,2*S*) β-bisabolol (40 m l^-1^) and bisabolene (0.6 mg l^-1^) on swarming motility of *S*. *marcescens*. *P* < 0.05, n = 3.

Since we observed a strong effect of *S*. *commune* volatiles on the tested indicator fungi, we next investigated whether also bacteria might response to fungal volatiles. Despite the absence of direct contact, *S*. *commune thn* strains exerted a strong growth reduction in *P*. *fluorescens and E*. *amylovora;* again no effect was observed using wildtype *S*. *commune* VOCs (Fig 3). Only slight inhibition was scored for *S*. *marcescens* (S5 Fig in S1 File). However, specifically with the latter bacterium, a reduced swarming activity was scored (Fig 4), which might impact colonization of timber. Again, these effects were more prominent when *S*. *marcescens* has been exposed to volatiles of the *thn* mutant strains of *S*. *commune* as compared to the wildtype monokaryotic strain.

### Quantification of sesquiterpenes released by *S*. *commune*

**Fig 5:**
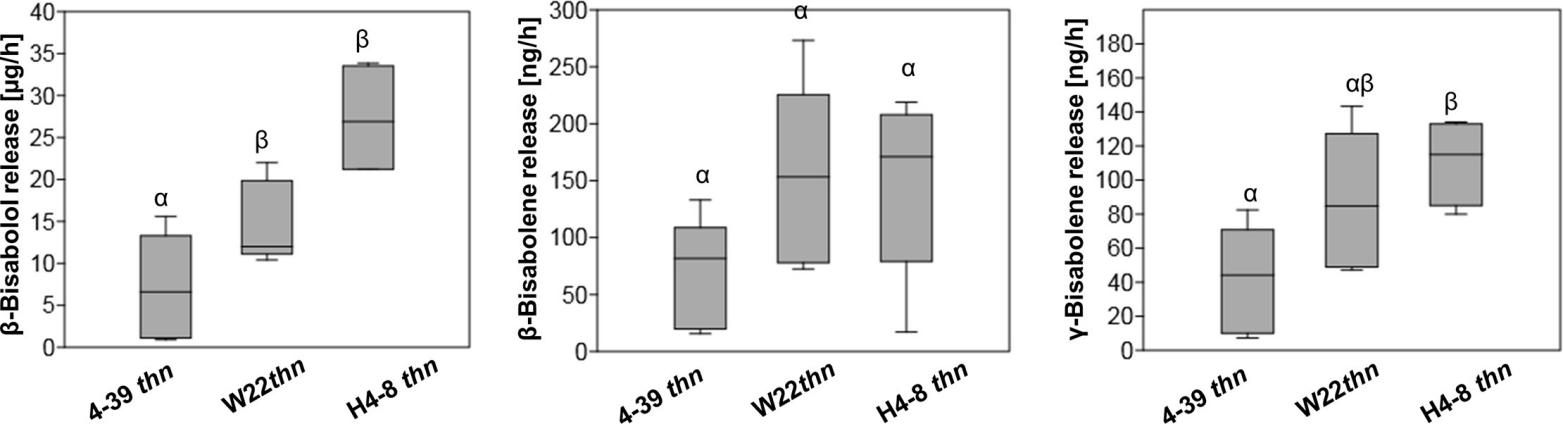
Quantification of sesquiterpenes from *S*. *commune thn* colonies pre-grown for 5 days on CYM. *P* < 0.05, n = 5.

**Fig 6:**
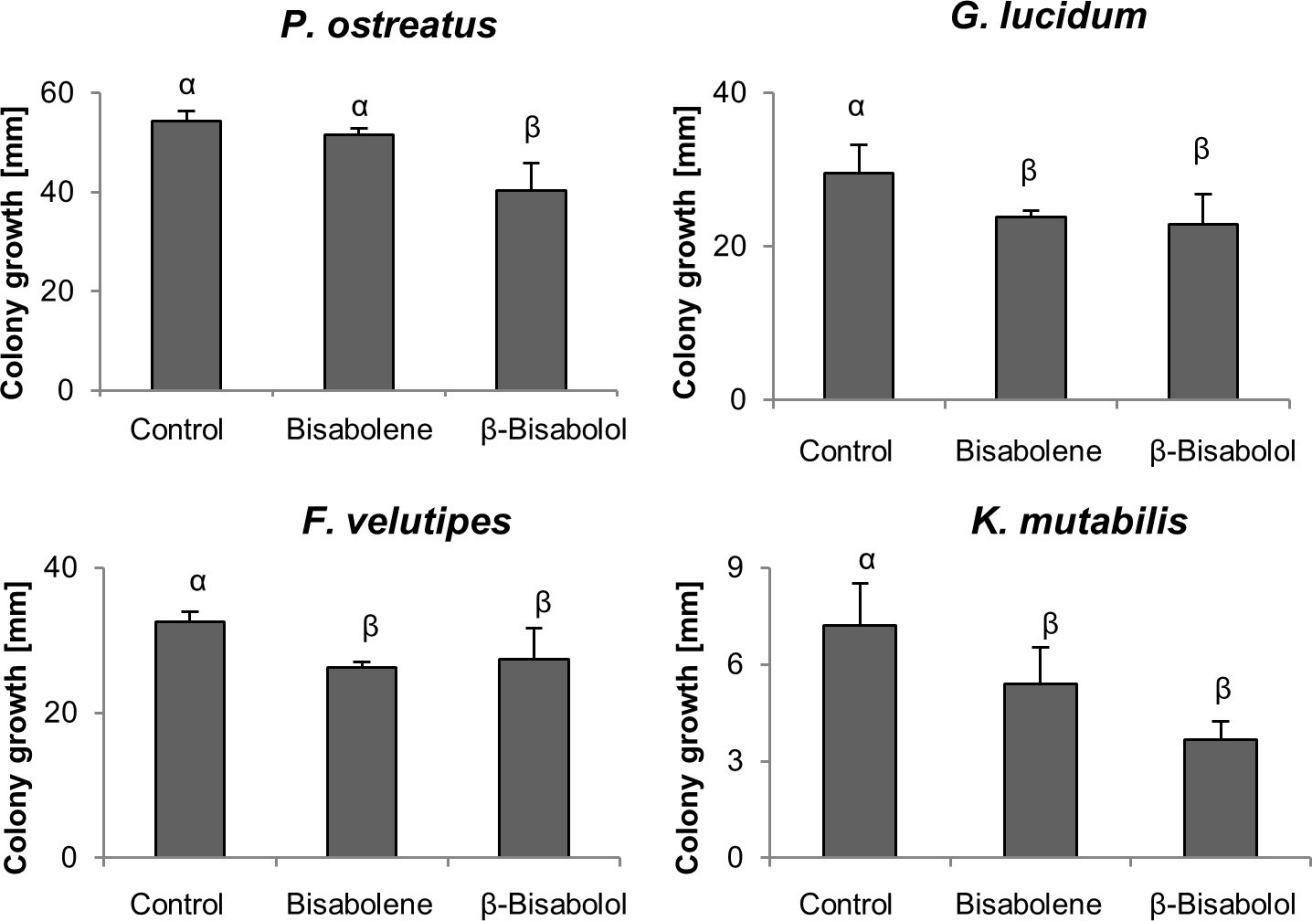
Effect of sesquiterpenes (-)-(1*R*,2*S*) β-bisabolol (40 mg l^-1^) and bisabolene (0.6 mg l^-1^) on the growth of indicator fungi. *P* < 0.05, n = 3 to 5.

The sesquiterpenes β-bisabolene, (*E*)-γ-bisabolene and (-)-(1*R*,2*S*)-β-bisabolol) were dominant in gas chromatography-mass spectrometry analysis of the headspace of *S*. *commune*. With respect to the *S*. *commune thn* strains, β-bisabolene was not significantly different between the mutant strains, but significant differences in the (-)-(1*R*,2*S*)-β-bisabolol and (*E*)-γ-bisabolene production were detected (Fig 5). While the strains *S*. *commune* H4-8 *thn* and *S*. *commune* W22 *thn* showed higher (-)-(1*R*,2*S*)-β-bisabolol abundance (27.2 ± 6.9 and 14.8 ± 4.9 *vs* 7.1 ± 6.3 μg h^-1^, *P* < 0.05), *S*. *commune* 4–39 *thn* headspace showed higher accumulation of (*E*)-γ-bisabolene (111.0 ± 25.6 *vs* 41.2 ± 31.6 ng h^-1^, *P* = 0.033). The enhanced inhibition of fungal growth by *S*. *commune* W22 *thn* coincides with the highest (-)-(1*R*,2*S*)-β-bisabolol amounts (Fig 6). From the sesquiterpene quantification of accumulation levels over the incubation time, adequate concentrations were determined in order to test effects on the growth of indicator bacteria and fungi.

### Effects of sesquiterpenes on fungal and bacterial growth

The presence of sesquiterpenes in the volatile profile of *S*. *commune thn* mutants was considered potentially responsible for the growth inhibition of the tested fungi and bacteria, because their absence in *S*. *commune* wildtype monokaryon correlated with reduced effects. The lack of an authentic standard prompted us to extract (-)-(1*R*,2*S*)-β-bisabolol directly from *S*. *commune thn* cultures, while for β-bisabolene and (E)-γ-bisabolene, a synthetic bisabolene mixture was available. We tested the sesquiterpenes for their antimicrobial activity, with the bisabolene mixture added at a concentration corresponding to a 6 day co-cultivation period. This led to growth inhibition in *G*. *lucidum*, *F*. *velutipes* and *K*. *mutabilis* (see Fig 6). The influence of (-)-(1*R*,2*S*)-β-bisabolol was even stronger, and a growth reduction for all tested fungi was observed.

With respect to the tested bacteria, neither (-)-(1*R*,2*S*)-β-bisabolol, nor the mixture of bisabolenes had an effect on bacterial growth that would mimic the effect of *S*. *commune thn* volatiles (S6 Fig in S1 File). Thus, additional VOCs must be necessary for the effect exerted on bacterial growth. However, the bisabolene mix as well as (-)-(1*R*,2*S*)-β-bisabolol significantly reduced swarming motility in *S*. *marcescens* (see Fig 4).

## Discussion

*S*. *commune* is adapted to the early colonization of wood infecting dead and living trees by utilizing enzymes that have been described for an intermediate wood decay mechanism between brown and white rot [32]. Here, we show that beneath fruiting bodies of *S*. *commune*, a specific microbial community was present that is associated with the wood degradation process. The presence of *S*. *commune* was confirmed, dominating the zone of wood decay and suppressing other fungi. Thus, *S*. *commune* likely could influence other members of the community. The co-occurring bacterial community represented presumably adapted strains. As a means of influencing the community structure, volatiles were identified.

As a means of study, *thn* mutants producing higher amounts of (-)-(1*R*,2*S*) β-bisabolol [22] were used to test for specific effects in interspecific interactions proven by pure sesquiterpene compounds. The composition of the volatile profile depends on growth condition and is highly variable during culture age, most likely due to shifts in the metabolism [33]. The production of sesquiterpenes and C8-volatiles seems to be linked to mating involving the signature compounds 1-octen-3-ol and 3-octanone, as well as (-)-(1*R*,2*S*) β-bisabolol for *S*. *commune* dikaryons [21]. Thus, sesquiterpenes are relevant for the local microbial community.

Sesquiterpenes are known fungal VOCs, and wood decay fungi, including *Lentinus lepideus*, *Polyporus pinicola*, *Resinicium bicolor*, *H*. *fasciculare* have been shown to produce sesquiterpenes [9, 16, 34]. Moreover, several plant species release a complex mixture of terpenes, including monoterpenes, sesquiterpenes, and diterpenes in response to wounding representing an important defense strategy or communication signal [35]. *Matricaria recutita* contains high amounts of α-bisabolol and extracts are used traditionally as herbal medicine against common human ailments [36]. In fungi the antimicrobial properties of sesquiterpenes were linked to defense response [10, 16], and other VOCs, like C8 volatiles, benzaldehyde and benzoic acid were shown to be involved in intra-kingdom and inter-domain interactions [37]. Subtle changes in growth rate, morphological alterations and pigment production can effect community composition [16]. We could show a strong decrease in mycelia growth, accompanied by a reduced formation of aerial mycelium, with *P*. *ostreatus*. In *Fusarium oxysporum* not only inhibiting the growth of antagonistic fungi, but also affecting aerial mycelial growth, modulating mycelial surface hydrophobicity, and influencing the expression of virulence genes could be associated with the sesquiterpenes α-humulene and β-caryophyllene [18]. The growth inhibition was also strong with *K*. *mutabilis*, while *G*. *lucidum* was less effected, in line with the strain specific (-)-(1*R*,2*S*) β-bisabolol and (*E*)-γ-bisabolene production. In the natural environment, fungi are not completely enclosed within the substrate and sesquiterpenes may not reach the concentrations measured in this study. However, individual sesquiterpenes were tested in appropriate doses to allow for comparison with co-cultivation experiments. The sesquiterpenes (-)-(1*R*,2*S*) β-bisabolol and (*E*)-γ-bisabolene inhibited the growth of all tested fungi, except for *P*. *ostreatus* being sensitive only to (-)-(1*R*,2*S*) β-bisabolol, which is in line with species specific susceptibility to VOCs [17].

Essential oils from plants containing sesquiterpenes show an antimicrobial activity against a wide range of bacterial species, which can be related to their ability to disrupt the structure of microbial cell membrane [36]. Also volatile-mediated interactions between soil bacteria and fungi have been described [10, 38]. In our study, bacteria seemed to be less impaired by *S*. *commune* volatiles, and the responsible compound impairing growth of *P*. *fluorescens* and *E*. *amylovora* could not be identified. Here additional VOCs, like esters and ketones could be relevant [22]. *S*. *marcescens* was not affected in growth, but showed lower motility, also when exposed to individual sesquiterpenes. This is in accordance with terpenes manipulating bacterial biofilm formation and motility [25]. The sesquiterpene alcohol α-bisabolol has been reported to inhibit prodigiosin pigment synthesis, biofilm formation and swarming motility in *S*. *marcescens* [39]. Swarming motility is controlled by quorum sensing and involves developmental modifications as well as metabolic changes [40], suggesting that sesquiterpenes interfere in the highly regulated cell-to-cell-communication process in bacteria.

Although pigmentation has been reported for VOC-mediated interaction [9, 16], this seems not the case for *S*. *commune* where direct physical contact was found to be prerequisite for pigment production [12]. Sesquiterpenes are lipophilic, targeting the membrane, and they can facilitate the passage of other toxins through membranes leading to a synergistic effect [35]. This would indicate that also membrane stability control could account for species specificity.

The tested sesquiterpenes strongly inhibited the growth of antagonistic fungi, reinforcing our hypothesis that sesquiterpenes contribute to the observed dominance of *S*. *commune* on infected wood. A high bacterial diversity was seen in early wood decay, with Proteobacteria dominating. The often restricted nitrogen availability in decayed wood can be overcome by the help of nitrogen-fixing bacteria, like *Rhizobium giardinii*, which has been detected in the community [41]. A high abundance of Alpha- and Gammaproteobacteria, Actinobacteria, Chitinophagia and Cytophagia was found inhabiting this habitat. A similar community composition has been reported before in decayed pine wood [41] and these phyla were also affiliated with the white-rot fungus *H*. *fasciculare* [3]. We observed a dominance of *Pseudomonadaceae* and *Sphingomonadaceae*, which are saprophytic plant colonizing bacteria that are known to improve plant growth by outcompeting pathogens, and they might be also be involved in lignocellulose degradation [4, 42]. Acidobacteria are well adapted to acidic conditions and were shown as being abundant in decaying wood [3, 41]. However, in our study members of this phylum were underrepresented in these samples.

Interestingly, we found comparatively high numbers of OTUs of Actinobacteria in the mycosphere of *S*. *commune*. These Gram-positive bacteria are predominately found in soil and are known for their ability to colonize wood and—at least partially—degrade lignin and cellulose [4, 41]. We isolated mostly wood colonizing Actinobacteria, including *Janibacter* sp., *Frigoribacterium* sp. and *Curtobacterium* sp., which has been described as a fungal growth promoting bacterium [43].

The cultivation provided conditions selecting for fast growing bacteria and fungi [44], which explains the differences of community analyses between the two methods. Nevertheless, both methods show different bias and it is essential to compare the results to obtain comprehensive insight into community structures. In addition, in order to test strains co-occurring with *S*. *commune*, we needed isolates from the same piece of wood. In conclusion, *S*. *commune* suppression of growth of competing species likely led to a selection for adapted microorganisms [3] that might enhance species depending on metabolic cross-feeding or other dependencies on co-occurring species involved in wood decay [45]. The reduce bacterial swarming motility of *S*. *marcescens* might also be a selective pressure, since swarming is a means to colonize new wood surfaces.

Thus we could highlight sesquiterpenes for their impact on community structure in the wood mycosphere.

## Supporting information

S1 File (PDF)
