# Supplementary material for: Function of sesquiterpenes from Schizophyllum commune in interspecific interactions
Source: PLoS One. 2021 Jan 15;16(1):e0245623. doi: 10.1371/journal.pone.0245623 (PMC7810277; doi:10.1371/journal.pone.0245623)
Supplement: S1 File — (PDF) [file pone.0245623.s001.pdf]

Supporting Information

S1 Table: Origin of indicator strains.

Species	Origin	Reference
**Fungi**		
Flammulina velutipes MG091201_01, FSU10496	Wood, Jena, Germany	IPF culture collection Frankfurt/M, Germany
Ganoderma lucidum MG100717_01, FSU:SF:013592	Wood, Königsfeld, Germany	IPF
Pleurotus ostreatus MG091105_01, FSU:9962	Wood, Jenaprießnitz, Germany	IPF
Kuehneromyces mutabilis MG091108_02, FSU:9992	Wood, Göttingen, Germany	IPF
**Bacteria**		
Pseudomonas fluorescens DSM-50090, JMRC:ST036370	United Kingdom	Leibniz Institute DSMZ – German Collection of microorganisms and cell cultures
Erwinia amylovora S59/5, DSM-30165, JMRC:ST:035608	*Pyrus communis*, United Kingdom	DSMZ
Serratia marcescens SM1, JMRC:ST036371	Germany	DSMZ

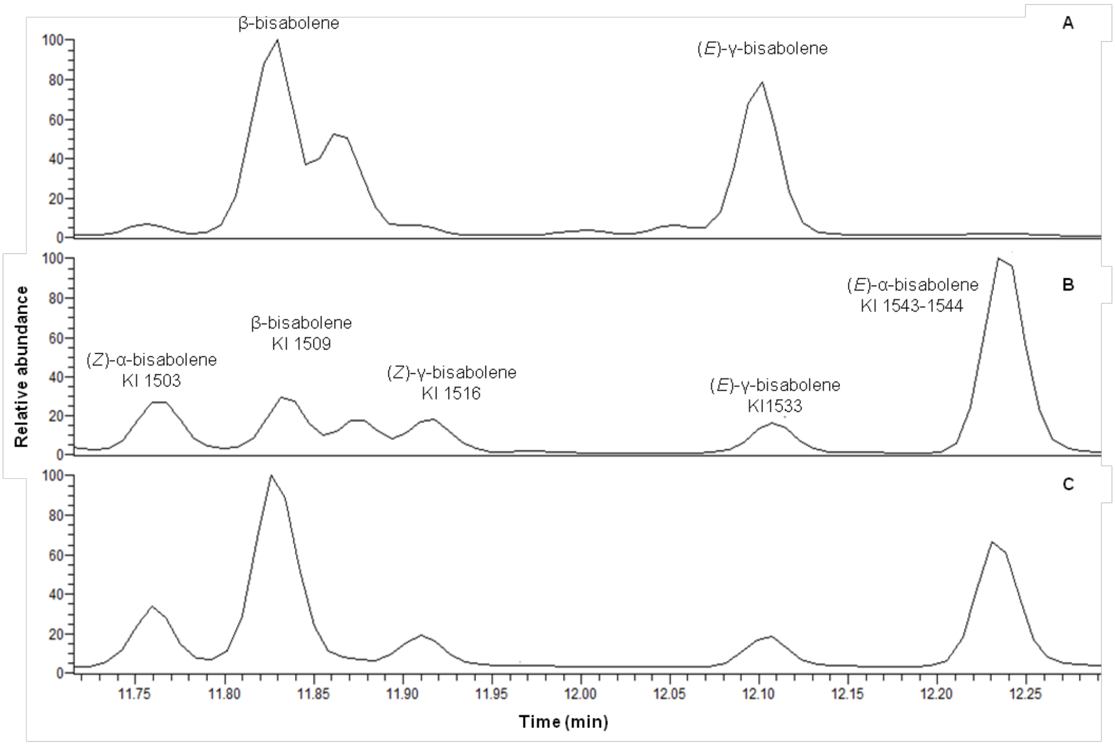

S1 Fig. Identification of bisabolenes by comparison with Kovats retention indices (KI). Chromatograms of bisabolene fraction of (A) *S. commune thn* strain, (B) bisabolene mixture (Alfa Aesar GmbH & Co KG, Karlsruhe, Germany), (C) synthetic bisabolene reference standard.

S2 Table. Composition of bacterial community in wood colonized by *S. commune.*

Phylum	Class	Family	Genus	Reads
**Acidobacteria**	Acidobacteriia	*Acidobacteriaceae*	*Acidobacterium*	167
	Blastocatellia	*Blastocatellaceae*	*Blastocatella*	19
	Holophagae	*Holophagaceae*	*Holophaga*	52
**Actinobacteria**	Acidimicrobiia	*Acidimicrobiaceae*	*Acidimicrobium*	25
			Ilumatobacter	24
		Iamiaceae	*Iamia*	40
	Actinobacteria	*Dermabacteraceae*	*Brachybacterium*	18
		Geodermatophilaceae	*Blastococcus*	86
		Kineosporiaceae	*Kineococcus*	17
		Microbacteriaceae	*Pseudoclavibacter*	54
			*Frigoribacterium**	51
		Micrococcaceae	*Arthrobacter*	285
		Micromonosporaceae	*Micromonospora*	242
		Nocardiaceae	*Rhodococcus*	19
		Nocardioidaceae	*Nocardioides*	44
			Friedmanniella	34
			Nocardioides	23
			Marmoricola	20
		Pseudonocardiaceae	*Actinomycetospora*	48
			Alloactinosynnema	27
			Amycolatopsis	19
		Streptomycetaceae	*Streptomyces*	67
		Thermomonosporaceae	*Actinomadura*	23
	Thermoleophilia	*Conexibacteraceae*	*Conexibacter*	56
		Solirubrobacteraceae	*Solirubrobacter*	27
**Bacteroidetes**	Bacteroidia	*Marinilabiliaceae*	*Anaerophaga*	20
	Chitinophagia	*Chitinophagaceae*	*Chitinophaga*	107
			Niastella	70
			Sediminibacterium	39
			Segetibacter	26
		Chloroflexaceae	*Chloroflexus*	18
	Cytophagia	*Cytophagaceae*	*Ohtaekwangia*	41
			Flexibacter	27
			Cytophaga	25
		Hymenobacteraceae	*Hymenobacter*	54
	Saprospiria	*Haliscomenobacteraceae*	*Haliscomenobacter*	27
	Sphingobacteriia	*Sphingobacteriaceae*	*Pedobacter*	233
			Mucilaginibacter	95
**Chloroflexi**	Dehalococcoidia	*Dehalococcoidaceae*	*Dehalococcoides*	22
	Thermomicrobia	*Sphaerobacteraceae*	*Sphaerobacter*	39
**Firmicutes**	Bacilli	*Carnobacteriaceae*	*Carnobacterium*	70
	Clostridia	*Eubacteriaceae*	*Acetobacterium*	41
			Eubacterium	32
**Gemmatimonadetes**	Gemmatimonadetes	*Gemmatimonadaceae*	*Gemmatimonas*	124
**Nitrospirae**	Nitrospira	*Nitrospiraceae*	*Nitrospira*	32
**Planctomycetes**	Phycisphaerae	*Phycisphaeraceae*	*Phycisphaera*	17
**Proteobacteria**	Acidithiobacillia	*Acidithiobacillaceae*	*Acidithiobacillus*	50
	Alphaproteobacteria	*Acetobacteraceae*	*Acidisphaera*	48
			Stella	23
			Saccharibacter	22
		Beijerinckiaceae	*Beijerinckia*	28
			Methyloferula	22
		Bradyrhizobiaceae	*Bradyrhizobium*	71
		Caulobacteraceae	*Brevundimonas*	67
			Phenylobacterium	27
			Caulobacter	50
		Erythrobacteraceae	*Erythrobacter*	18
		Hyphomicrobiaceae	*Rhodoplanes*	100
			Hyphomicrobium	59

			Devosia	93
			Ancalomicrobium	24
			Pedomicrobium	19
			Hirschia	20
		Methylobacteriaceae	*Methylobacterium*	93
		Phyllobacteriaceae	*Mesorhizobium*	45
		Rhizobiaceae	*Agrobacterium*	60
			Rhizobium	23
		Rhodobacteraceae	*Rhodobacter*	34
		Rhodobiaceae	*Parvibaculum*	38
		Rhodospirillaceae	*Oceanibaculum*	45
			Pelagibius	31
			Magnetovibrio	20
		Sphingomonadaceae	*Sphingomonas*	541
			Kaistobacter	24
			Novosphingobium	40
			Sphingopyxis	18
		Xanthobacteraceae	*Pseudolabrys*	18
	Betaproteobacteria	*Burkholderiaceae*	*Burkholderia*	133
			Cupriavidus	78
		Comamonadaceae	*Hydrogenophaga*	72
			Curvibacter	26
		Gallionellaceae	*Sideroxydans*	43
		Hydrogenophilaceae	*Thiobacillus*	37
		Methylophilaceae	*Methylotenera*	29
		Nitrosomonadaceae	*Nitrosospira*	118
		Oxalobacteraceae	*Massilia*	313
			Duganella	53
			Herbaspirillum	43
			Noviherbaspirillum	21
		Rhodocyclaceae	*Denitratisoma*	125
			Dechloromonas	32
		Unclassified family	*Aquabacterium*	87
			Methylibium	27
			Ideonella	26
			Leptothrix	17
	Deltaproteobacteria	*Anaeromyxobacteraceae*	*Anaeromyxobacter*	19
		Desulfobacteraceae	*Desulfobacterium*	30
		Desulfobulbaceae	*Desulfobulbus*	21
		Geobacteraceae	*Geobacter*	110
		Kofleriaceae	*Haliangium*	29
		Polyangiaceae	*Chondromyces*	22
	Gammaproteobacteria	*Alteromonadaceae*	*Marinobacte*	26
			Alishewanella	17
		Chromatiaceae	*Thiorhodovibrio*	38
		Ectothiorhodospiraceae	*Thiorhodospira*	46
		Enterobacteriaceae	*Raoultella*	5223
			Gibbsiella	55
			Buttiauxella	32
			Enterobacter	22
		Erwiniaceae	*Erwinia*	19006
			Pantoea	3448
		Methylococcaceae	*Methylomonas*	30
			Methylomicrobium	28
			Methylobacter	20
		Microbulbiferaceae	*Microbulbifer*	29
		Moraxellaceae	*Acinetobacter*	88
			Perlucidibaca	41
			Alkanindiges	18
		Oceanospirillaceae	*Marinomonas*	18
		Oleiphilaceae	*Oleiphilus*	29

		Piscirickettsiaceae	*Methylophaga*	36
		Pseudomonadaceae	*Pseudomonas**	76294
			*Luteibacter**	863
		Sinobacteraceae	*Alkanibacter*	146
			Solimonas	139
			Steroidobacter	76
			Nevskia	39
		Thioalkalispiraceae	*Thiohalophilus*	70
			Thioalkalispira	66
		Thiotrichaceae	*Thiothrix*	132
			Beggiatoa	61
		Xanthomonadaceae	*Thermomonas*	137
			Pseudoxanthomonas	119
			Lysobacter	27
			Xylella	21
		Yersiniaceae	*Rahnella*	46054
		Unclassified family	*Alkalimonas*	43
	Zetaproteobacteria	*Mariprofundaceae*	*Mariprofundus*	1016
**Tenericutes**	Mollicutes	*Acholeplasmataceae*	*Candidatus*	25
**Verrucomicrobia**	Opitutae	*Opitutaceae*	*Opitutus*	22
	Verrucomicrobiae	*Verrucomicrobiaceae*	*Prosthecobacter*	81

* Cultivated under laboratory conditions

S3 Table. Composition of fungal community in wood colonized by *S. commune.*

Phylum	Class	Family	Genus	Reads
**Ascomycota**	Dothideomycetes	*Cladosporiaceae*	*Cladosporium*	10
		Didymellaceae	*Didymella*	2
			Peyronellaea	2
			Phoma	324
		Leptosphaeriaceae	*Leptosphaeria*.	3
		Phaeosphaeriaceae	*Chaetosphaeronema*	2
		Pleosporaceae	*Alternaria*	2
			Curvularia	2
		Sporormiaceae	*Preussia*	2
		Unclassified family	*Ochrocladosporium*	2
	Eurotiomycetes	*Aspergillaceae*	*Penicillium*	2
		Herpotrichiellaceae	*Exophiala*	6
	Leotiomycetes	*Rhytismataceae*	*Rhytisma*	24
		Unclassified family	*Leptodontidium*	2
	Pezizomycetes	*Pyronemataceae*	*Wilcoxina*	3
		Tuberaceae	*Tuber*	8
	Saccharomycetes	*Saccharomycodaceae*	*Hanseniaspora*	112
	Sordariomycetes	*Coniochaetaceae*	*Coniochaeta*	528
		Cordycipitaceae	*Isaria*	5
		Nectriaceae	*Ilyonectria*	2
		Ophiocordycipitaceae	*Ophiocordyceps*	4
		Stachybotryaceae	*Myrothecium*	2
		Togniniaceae	*Phaeoacremonium*	294
		Unclassified family	*Sarocladium*	9
**Basidiomycota**	Agaricomycetes	*Amanitaceae*	*Amanita*	17
		Boletaceae	*Boletellus*	6
		Bondarzewiaceae	*Heterobasidion*	3
		Ceratobasidiaceae	*Ceratobasidium*	4
			Rhizoctonia	3
		Cortinariaceae	*Flammulaster*	22
		Hygrophoraceae	*Hygrocybe*	6
		Schizophyllaceae	*Schizophyllum**	34350
		Serendipitaceae	*Serendipita*	47
		Thelephoraceae	*Tomentella*	5
	Agaricostilbomycetes	*Kondoaceae*	*Bensingtonia*	6
	Tremellomycetes	*Bulleribasidiaceae*	*Mingxiaea*	21

		Sirobasidiaceae	*Fibulobasidium*	5
		Tremellaceae	*Tremella*	2
**Chytridiomycota**	Chytridiomycetes	*Rhizophydiaceae*	*Rhizophydium*	22
	Monoblepharidomycetes	Unclassified family	*Monoblepharella*	3
	Unclassified	Unclassified family	*Paramicrosporidium*	14
	Unclassified	*Olpidiaceae*	*Olpidium*	3
**Glomeromycota**	Glomeromycetes	*Diversisporaceae*	*Redeckera*	2
		Glomeraceae	*Glomus*	9
		Paraglomeraceae	*Paraglomus*	19
		Unclassified family	*Entrophospora*	3
**Neocallimastigomycota**	Neocallimastigomycota	*Neocallimastigomycota*	*Anaeromyces*	3
**Zygomycota**	Zygomycetes	*Basidiobolaceae*	*Basidiobolus*	4
	Unclassified	*Mortierellaceae*	*Mortierella*	25

* Cultivated under laboratory conditions

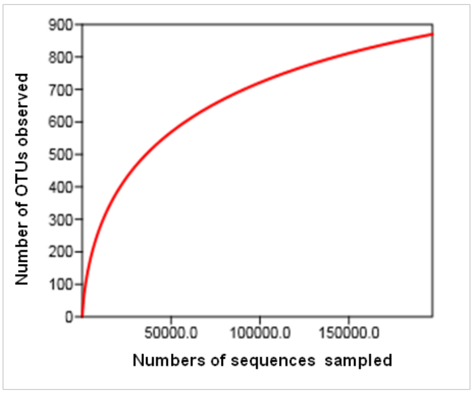

S2 Fig. Rarefaction curve for the microbial community in the mycosphere of *S. commune.*

S4 Table. Community characteristics and α-diversity indices.

	Bacterial community	Fungal community
Final high quality read pairs	162,737	35,984
Filtered high quality read pairs	160,145	35,956
OTUs	165	54
H_S	2.249	0.288
E_h	0.057	0.0247
D	0.173	0.913
H_{1-D}	0.827	0.087
Chao1	165	122

OTUs - operational taxonomic unit, H_S – Shannon's diversity index, E_h – Shannon's equitability, D – Dominance = 1-Simpson's index, H_{1-D} – Simpson's index

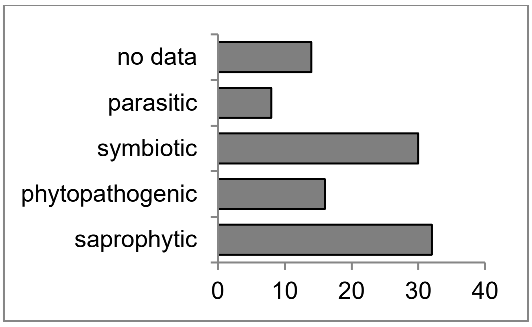

S3 Fig. Different lifestyles (%) of the members of the fungal community.

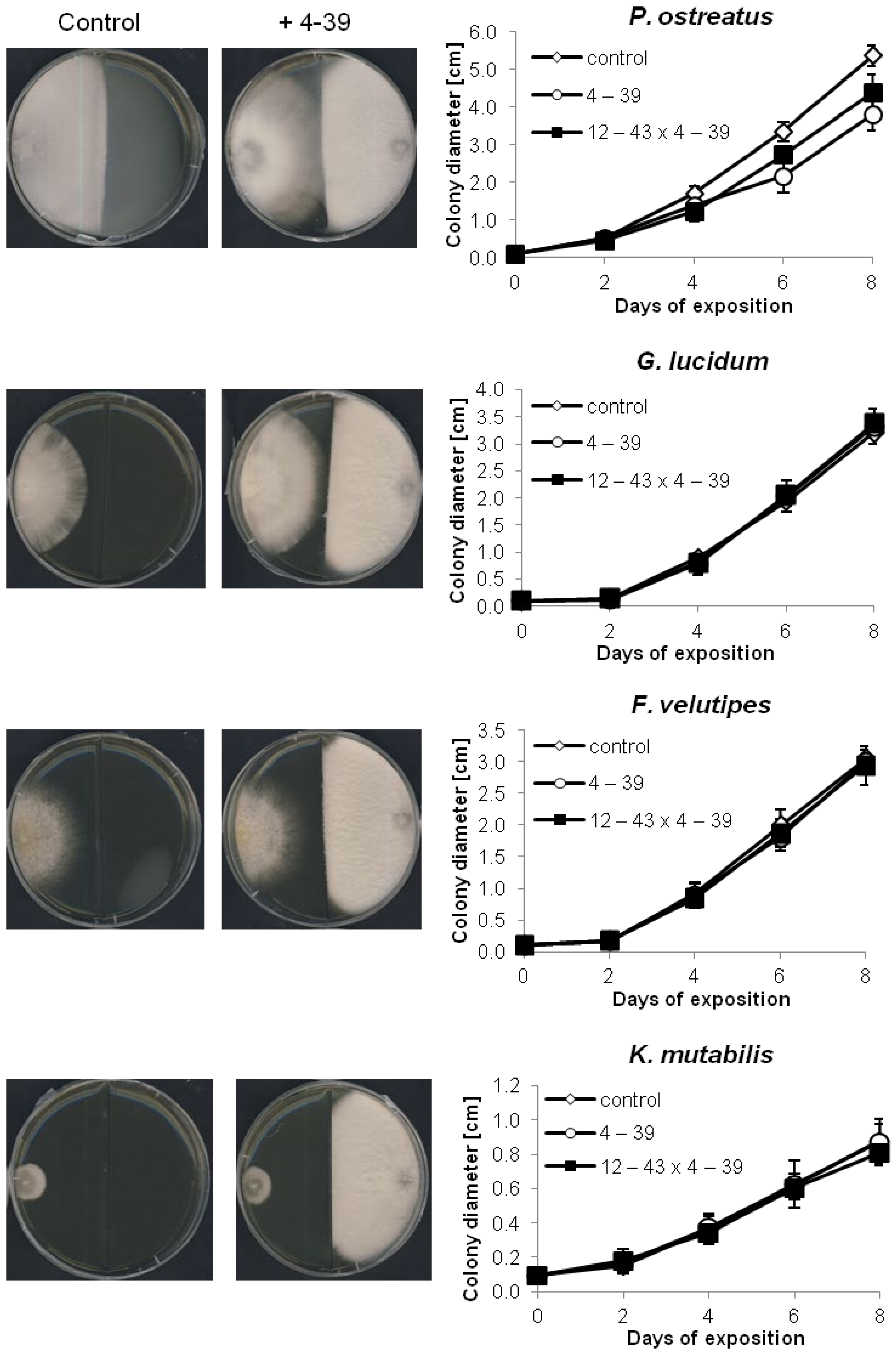

S4 Fig. Effect of *S. commune* volatile organic compounds (VOCs) on the growth of growth of indicator fungi. n = 10.

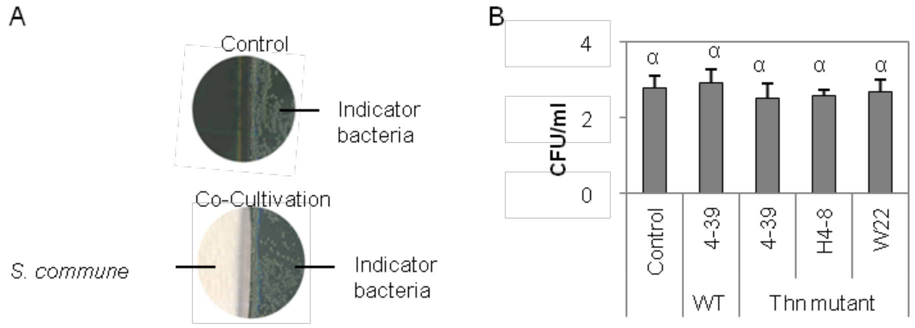

S5 Fig. Effect of volatiles produced by *S. commune* wildtype and *thn* mutant strains on the growth of indicator bacteria. (A) Indicator bacteria were grown alone (control) or besides *S. commune* strains (co-cultivation) on StdI medium. Growth of *S. marcescens* (B) cultivated in the presence of *S. commune.* Data are presented as viable cell numbers (CFU ml^{-1}) in 10^8*;* $P < 0.05$ (n = 6).

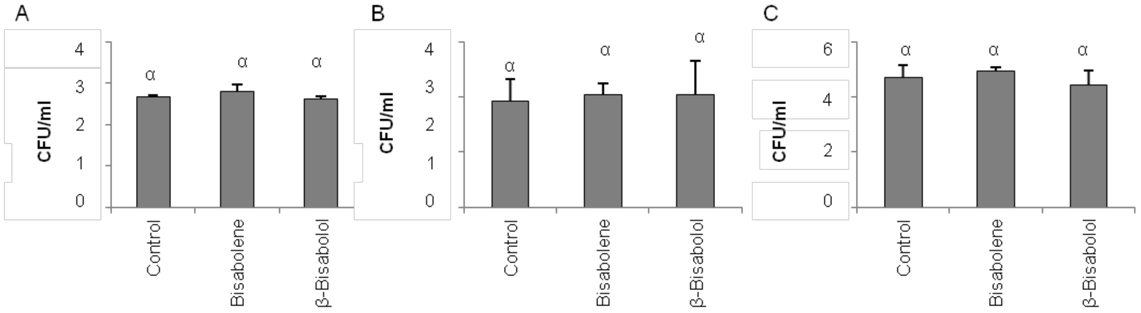

S6 Fig. Effect of sesquiterpenes on the growth of indicator bacteria. (-)-(1*R*,2*S*) β-Bisabolol (40 mg l^{-1}) and bisabolene (0.6 mg l^{-1}) did not affect the growth of *S. marcescens* (A) and *P. fluorescens* (B) and *E. amylovora* (C). Data are presented as viable cell numbers (CFU ml^{-1}) in 10^8; $P < 0.05$, n=3.
